# In a secondary analysis from a randomised, double-blind placebo-controlled trial Dexmedetomidine blocks cholinergic dysregulation in delirium pathogenesis in patients with major surgery

**DOI:** 10.1038/s41598-023-30756-z

**Published:** 2023-03-09

**Authors:** Yanite Jacob, Bill Schneider, Claudia Spies, Maria Heinrich, Clarissa von Haefen, Widuri Kho, Anne Pohrt, Anika Müller

**Affiliations:** 1 Department of Anesthesiology and Operative Intensive Care Medicine (CCM, CVK), Charité - Universitätsmedizin Berlin, corporate member of Freie Universität Berlin, Humboldt-Universität Zu Berlin, and Berlin Institute of Health, Charité Platz 1, 10117 Berlin, Germany; 2 Berlin Institute of Health (BIH), Anna-Louisa-Karsch 2, 10178 Berlin, Germany; 3 Institute of Biometry and Clinical Epidemiology, Charité - Universitätsmedizin Berlin, corporate member of Freie Universität Berlin, Humboldt-Universität Zu Berlin, Berlin, Germany

**Keywords:** Molecular medicine, Randomized controlled trials, Geriatrics

## Abstract

Dexmedetomidine is an alpha-2 adrenoreceptor agonist with anti-inflammatory and anti-delirogenic properties. Pathogenesis of postoperative delirium (POD) includes cholinergic dysfunction and deregulated inflammatory response to surgical trauma. Acetylcholinesterase (AChE) and butyrylcholinesterase (BChE) are discussed as biomarkers for both POD and severity in acute inflammation. To show whether there is a link between blood cholinesterase activities and dexmedetomidine, we performed a secondary analysis of a randomised, double-blind, placebo-controlled trial that recently showed a lower incidence of POD in the dexmedetomidine group. Abdominal or cardiac surgical patients aged ≥ 60 years were randomised to receive dexmedetomidine or placebo intra- and postoperatively in addition to standard general anaesthesia. We analysed the course of perioperative cholinesterase activities of 56 patients, measured preoperatively and twice postoperatively. Dexmedetomidine resulted in no change in AChE activity and caused a rapid recovery of BChE activity after an initial decrease, while placebo showed a significant decrease in both cholinesterase activities. There were no significant between-group differences at any point in time. From these data it can be assumed that dexmedetomidine could alleviate POD via altering the cholinergic anti-inflammatory pathway (CAIP). We advocate for further investigations to show the direct connection between dexmedetomidine and cholinesterase activity.

## Introduction

Dexmedetomidine is a highly selective alpha-2-adrenoreceptor agonist with specific sympatholytic effects that is commonly used for sedation, analgesia, and anxiolysis in the intensive care unit^1,2^. Sedation is mediated without significant respiratory depression by decreasing brain activity in the locus coeruleus^1^. In the perioperative setting, dexmedetomidine has been shown to significantly reduce the need for hypnotics and opioids^3–5^. Common side effects include hypotension and bradycardia^6^. The recently published SPICE III trial showed that dexmedetomidine reduces 90-day mortality in elderly patients (over 65 years of age) compared with other sedation regimens, regardless of the reason for admission. In contrast, younger patients with nonoperative status had a high probability of increased 90-day mortality^7^. Furthermore, dexmedetomidine has anti-delirogenic^8–10^ and anti-inflammatory properties, the latter of which may attenuate an excessive inflammatory response after surgery^11^.

Dexmedetomidine has been shown to reduce the incidence of postoperative delirium (POD) when used during surgery or in the intensive care unit^8,12,13^. POD is characterized by disturbance of attention and awareness that starts acutely (e.g. after surgery) with a tendency to fluctuate, and at least one additional disturbance in cognition, and is associated with high mortality, ranging from 24 to 50%^14,15^. While the molecular mechanisms underlying the anti-deliriogenic potential of dexmedetomidine are still unknown, several anti-inflammatory mechanisms have already been described^16–24^, including the enhancement of the cholinergic anti-inflammatory pathway (CAIP)^25–28^. Indeed, one of the hypothesised molecular mechanisms of POD is the deregulation of the CAIP caused by cholinergic deficiency, leading to increased inflammation^29–33^.

The CAIP acts as an immunological reflex that links the nervous and immune systems. Consisting of the efferent part of the vagus nerve, the neurotransmitter acetylcholine (ACh), and the alpha 7 subunit of the nicotinic acetylcholine receptor (α7nAChR), the CAIP mediates the cholinergic regulation of inflammation. Following tissue damage or infection, it becomes activated by the release of pro-inflammatory cytokines and in turn controls the immune response via ACh, which binds to the α7nAChR on immune cells^34–36^. This ultimately leads to a dose-dependent inhibition of pro-inflammatory cytokine production (e.g., tumor necrosis factor alpha (TNFα), interleukin (IL)-1β and IL-6) by suppressing the activation of NF-kB particularly in the spleen^37–39^. In contrast, the synthesis and secretion of anti-inflammatory cytokines (e.g., IL-10) are maintained^39^.

In mice with LPS-induced inflammation, pre-emptive administration of dexmedetomidine attenuated NF-κB activation and the production of TNF-α, IL-6, and IL-1β at both the mRNA and protein levels in the spleen^40^. Consistent with these results, Kho et al. also found an effect of dexmedetomidine on cholinesterases in the spleen of rats^41^.

The acetylcholine hydrolysing enzymes acetylcholinesterase (AChE) and butyrylcholinesterase (BChE) degrade ACh. AChE is found in all excitable tissues, in most erythrocytes, in immune cells, and in placental tissue and hydrolyzes ACh with a very high affinity. AChE is inhibited at high acetylcholine concentrations. BChE (also called as “pseudo” or “plasma” cholinesterase) is found ubiquitously throughout the body, particularly in the liver, blood serum, pancreas and central nervous system. In the brain, BChE is located mainly in glial and endothelial cells. It hydrolyses with a lower affinity ACh and non-choline esters, and is not inhibited by higher ACh concentrations^42^.

The regulation of AChE and BChE is still the subject of ongoing research. However, previous results from cell cultures and animal experiments indicate that during inflammation, the expression and activity in particular of AChE is modulated in a cell-specific manner by a direct interaction with the α7nAChR, but also by the binding of NF-κB to the AChE promoter gene^43–45^.

An association between cholinesterase enzyme activity and the occurrence of POD has been proposed by several studies, suggesting that lower activity even preoperatively puts patients at risk for POD^46–51^. However, given the differences in perioperative activity profiles of cholinesterases, this remains controversial. In addition, there is evidence for an indicative role of cholinesterases in systemic inflammation^52–54^. In this regard, especially BChE activity seems to be a promising candidate for clinical use, because it correlates negatively with conventional inflammatory markers such as C-reactive protein (CRP), with shorter time of latency and rapid determination by point-of-care measurement^55–58^.

Dexmedetomidine is a particularly interesting drug in this context, as it has an anti-inflammatory effect on the one hand and an anti-delirogenic potential on the other. Moreover, to the best of our knowledge, there have been no clinical reports of a possible effect of dexmedetomidine on cholinesterase activities.

Based on all these considerations, we hypothesise that dexmedetomidine has an influence on peripheral cholinesterase activities, potentially related to its anti-delirogenic and anti-inflammatory effects. To examine this hypothesis, we used data from the NEUPRODEX trial, a double-blind randomised controlled trial, which showed that perioperative administration of dexmedetomidine resulted in a significantly lower incidence of POD^8^. Therefore, this secondary analysis investigates whether the use of dexmedetomidine in addition to general anaesthesia alters the perioperative course of AChE and BChE activity.

## Methods

### Study design and population

This is a secondary analysis of the prospective, randomised, double-blind placebo-controlled NEUPRODEX trial that took place between July 2014 and July 2018^8^. The primary outcome of this study was the postoperative incidence of delirium as measured by CAM-ICU (Confusion Assessment Method for the intensive care unit) or CAM (Confusion Assessment Method for normal wards) twice daily until the fifth postoperative day. The trial was approved by the regional ethics committee of Berlin, Germany (Landesamt für Gesundheit und Soziales, Approval Number 13/0491-EK 11) and was registered in the EU clinical trials register (2013–000,823-15) and the American National Institute of Health register (NCT02096068 on 26/03/2014). Inclusion and exclusion criteria are stated in our recent publication about the primary outcome as well as randomization process^8^. After written informed consent, the elderly patients were randomly assigned to dexmedetomidine or placebo group. The requirement for inclusion in this secondary analysis was the existence of a complete series of measurements of perioperative AChE and BChE activity. Patients with incomplete values were not included.

All patients received general anaesthesia induced with propofol and were maintained with either propofol or sevoflorane. All measures and procedures were performed in accordance with relevant guidelines and regulations. Our hospital pharmacy prepared syringes with dexmedetomidine or placebo, blinding both physicians and investigators. Starting ten minutes after induction, patients received either 0.7 µg/kg adjusted body weight (ABW)/h continuously or an equivalent volume of normal saline. The dose was reduced to 0.4 µg/kg ABW/h 30 min before the end of surgery. After arrival at the ICU, the dose was further reduced, but could be increased again to achieve a RASS score between 0 and − 1. During surgery, bradycardia as a side effect of dexmedetomidine was treated either by administration of orciprenaline or dose reduction of the syringe driver, containing either dexmedetomidine or placebo.

For analysis, we classified patients according to anticholinergic burden of their permanent medication taken at home. The Anticholinergic Drug Scale (ADS) developed by Carnahan and his colleagues^59^ with originally four items was dichotomized into 0 (no preoperative anticholinergic burden) and 1 (level of anticholinergic burden ≥ 1) to evaluate whether possible alterations of cholinesterase activity by these medications were balanced between groups.

### Baseline measurements

The following baseline measurements were analysed: age, sex, Body Mass Index (BMI), American Society of Anaesthesiology Physical Score (ASA PS), quantity of preoperative long-term agents taken by the patient, polypharmacy defined as more than five preoperative long-term agents, Anti-cholinergic Drug Scale (ADS) of the long-term agents, type of surgery, incision-suture time, applied volume of placebo or dexmedetomidine.

Complementary to the baseline characteristics, opioid, anaesthetic, and red blood cell transfusion requirements were recorded from the intraoperative data as potential influencing factors on cholinesterase activities.

### Cholinesterase activity

Cholinesterase activities were measured in all the whole blood samples drawn from arterial lines or venepuncture at three different points in time: preoperatively (on the evening before surgery or right before anaesthesia induction), 15 min after surgery, and the next morning after surgery at 8 a.m. (± 1 h). We analysed the samples with the portable point-of-care photometry test kit and machine “ChE check mobile” according to the instructions of the manufacture (SECURETEC, Neubiberg, Germany). This test kit applies a modified Ellmann reaction according to Worek et al. to determine the activity of AChE (normalized to haemoglobin U/g Hb) and BChE (U/l). The testing was performed immediately after collecting the blood sample.

In order to compare cholinesterase activities between delirium and no delirium, we first adjusted the delirium incidence for our observation period. Patients were classified as delirium positive if they were positively detected with the CAM/CAM-ICU at least once until the first postoperative day.

### Statistical analysis

Baseline and intraoperative characteristics were expressed as median and lower and upper quartile, or frequencies with percentages. Descriptive statistics were computed for the dexmedetomidine group and the placebo group, heterogeneity between groups was assessed using Chi^2^ tests and Mann–Whitney U tests. Changes in cholinesterase activities over time within the group were assessed using Friedman rank analysis and the differences at each time point were compared using Mann–Whitney U tests. All testing was two-sided using a significance level of 0.05 to indicate statistical significance. Analyses were conducted with SPSS 25 [IBM SPSS Statistics® Version 25, IBM Germany, Ehningen, Germany] for Macintosh.

### Institutional review board statement

The trial was approved by the regional ethics committee of Berlin, Germany (Landesamt für Gesundheit und Soziales, Approval Number 13/0491-EK 11) and was registered in the EU clinical trials register (2013-000823-15) and the American National Institute of Health register (NCT02096068).

## Results

**Figure 1:**
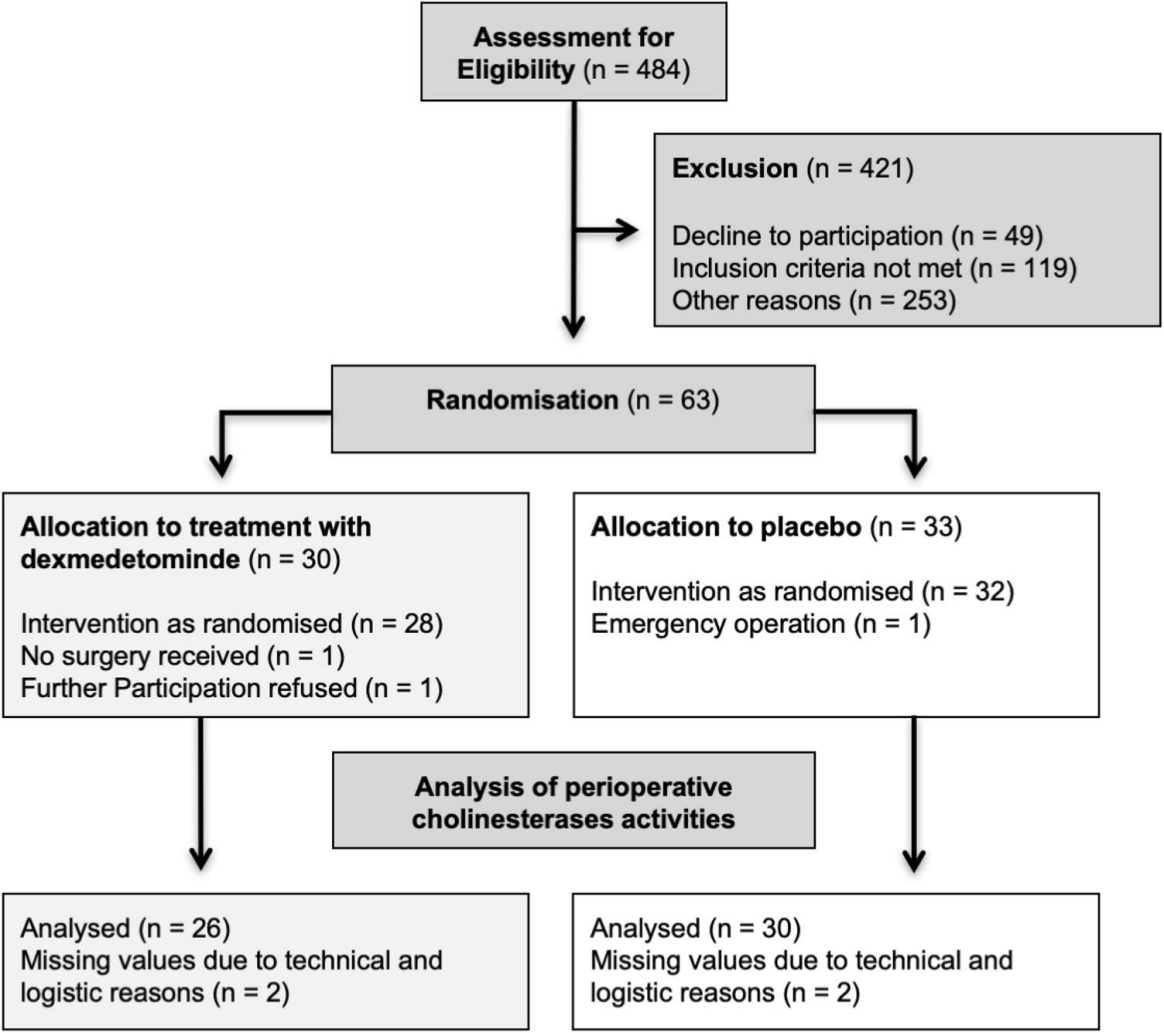
CONSORT flow chart.

We screened 484 patients for eligibility that were scheduled to receive either elective coronary artery bypass graft surgery with reduced left ventricular ejection fraction ≤ 30%, or major abdominal surgery. 63 patients could be included according to inclusion and exclusion criteria. Three patients withdrew from enrolment as explained in our previous publication, yielding 60 patients. After merging preoperative cholinesterase measurements from the day before surgery with surgery day values, 56 cases (median age 69 years, 29% female) of complete cholinesterase activity measurements could be included in the analyses, while four cases had to be excluded because of missing cholinesterase values (see flow chart, Fig. 1).

### Baseline characteristics did not differ between placebo and dexmedetomidine group

Baseline characteristics of the patients included in the present study differed slightly from our previously reported trial in the same population because four patients had to be excluded from analysis of cholinesterase activity, as stated in the methods^8^. Table 1 depicts the following baseline measurements. Of 56 patients included, 30 received standard general anaesthesia and 26 dexmedetomidine in addition. Median age did not differ significantly between the placebo group (69 years) and the dexmedetomidine group (71.5 years). Female patients were a minority in both groups, but the frequencies of female patients were not significantly different between placebo (30%) and dexmedetomidine (26.9%). There was also no significant difference between placebo and dexmedetomidine regarding Body Mass Index (median 27.1 m^2^/kg). About half of the population in both groups were patients with severe disease which limits activity (ASA PS III) or severe disease posing a constant threat to life (ASA PS IV). Most of the surgeries in both groups were abdominal (median 78.6%), only 21.4% were cardiac surgeries. Because preoperative medication can influence the cholinergic system, we analysed baseline values of the quantity of preoperative agents, the frequency of polypharmacy (intake of more than five different agents per day) and the number of cholinergic agents. Polypharmacy was frequent (55.4%) and 14.3% had at least one item on the ADS, with no significant differences between the two groups. Importantly, the applied volume of substance containing the agent in the dexmedetomidine group and the volume of placebo substance did not differ. Of interest, the baseline parameter with the lowest, yet not significant *P*-value was the incision-suture time. By tendency, it was faster in the dexmedetomidine (3.58 h) than in the placebo group (4.45 h).

**Table 1:** Baseline characteristics of 56 patients.

	All	Placebo	Dex	*P*-value
	n = 56 (100%)	n = 30 (53.6%)	n = 26 (46.4%)	
Age (years)				
Median [IQR]	69 [65–75.5]	69 [65–75]	71.5 [62–76]	0.928^b^
Sex				
Female	16 (28.6%)	9 (30%)	7 (26.9%)	0.799^a^
BMI, kg/m^2^				
Median [IQR]	27.1 [24.4–30.50]	27,29 [24.9–32.10]	26,78 [23.4–30]	0.465^b^
ASA PS				
III + IV	29 (51.8%)	15 (50%)	14 (53.8%)	0.774^a^
Quantity of preoperative agents				
Median [IQR]	5 [3–7]	5 [3.8–6]	5 [2.75-8]	0.710^b^
Polypharmacy with ≥ 5 preoperative agents				
ADS	31 (55.4%)	17 (56.7%)	14 (53.8%)	0.832^a^
≥ 1	8 (14.3%)	3 (11.1%)	5 (20%)	0.375^a^
Type of surgery				
Abdominal	44 (78.6%)	24 (75.0%)	20 (78.6%)	0.780^a^
Cardiac	12 (21.4%)	6 (25%)	6 (21.4%)	
Incision-suture time, hours				
Median [IQR]	4.31 [3–5.83]	4.45 [3.6–5.9]	3.58 [2.45–5.8]	0.135^b^
Applied volume of placebo/ Dex				
Median [IQR]	83.20 [51.9–115.5]	76.3 [59–111.6]	84.8 [46–123.6]	0.961^b^

Statistical analysis was conducted between patients receiving standard general anaesthesia (Placebo) and dexmedetomidine in addition to general anaesthesia (Dex).

*ASA PS* American Society of Anaesthesiology Physical Score, *BMI* Body Mass Index, *ADS* anticholinergic drug scale of preoperative long-term medications, *Dex* dexmedetomidine, *IQR* interquartile range.

^a^Categorical variables analysed by Chi^2^ test.

^b^Continuous variables analysed by Mann–Whitney U test.

### Intraoperative opioid, anaesthetic, and transfusion requirements are comparable in both groups

In addition to baseline characteristics, we evaluated intraoperative opioid, anaesthetic and transfusion requirements, as these factors may potentially influence cholinesterase activity. There was no difference found between the two groups in regard to these measurements (Table 2).

**Table 2:** Intraoperative opioid, anaesthetic and transfusion requirements of 56 patients.

	Placebo	Dex	*P*-value
	n = 30 (53.6%)	n = 26 (46.4%)	
Midazolam premedication			
n (%)	8 (26.7%)	5 (19.2%)	0.511^a^
Propofol bolus			
n (%)	29 (96.7%)	26 (100%)	0.732^a^
Cum.; mg; Median [IQR]	180 [150–200]	185 [140–200]	0.706^b^
TIVA with propofol			
n (%)	6 (20%)	9 (34.6%)	0.454^a^
mg/kg/h; Median [IQR]	3.77 [3.33–5.91]	3.65 [3.0–4.96]	0.680^b^
Sevoflurane			
n (%)	1.45 [1.25–1.75]	19 (73.1%)	0.426^a^
et vol%; Median [IQR]	21 (70.0%)	1.37 [1.15–1.55]	0.126^b^
Desflurane			
n (%)	10 (33.3%)	7 (26.9%)	0.386^a^
et Vol%; Median [IQR]	4.44 [4.10–4.81]	3.86 [3.45–4.55]	0.079^b^
Fentanyl			
n (%)	24 (80%)	20 (76.9%)	0.642^a^
Cum.; mg; Median [IQR]	0.5 [0.36–0.93]	0.44 [0.40–0.75]	0.661^b^
Sufentanile			
n (%)	6 (20.0%)	7 (26.9%)	0.369^a^
Cum.; µg; Median [IQR]	194 [102.53–298.73]	201 [100–235]	0.886^b^
Rocuronium			
n (%)	18 (60%)	15 (57.7%)	0.188^a^
Cum.; mg; Median [IQR]	67.5 [50–100]	90 [80–115]	0.061^b^
Cisatracurium			
n (%)	13 (43.3%)	11 (42.3%)	0.372^a^
Cum.; mg; Median [IQR]	17 [13–25.50]	15 [15–20]	0.600^b^
RBC Transfusion			
n (%)	4 (13.3%)	3 (11.5%)	0.421^a^
Cum.; ml; Median [IQR]	320 [308.5–744.75]	620 [310–620]	0.372^b^

Statistical analysis was conducted between patients receiving standard general anaesthesia (Placebo) and dexmedetomidine in addition to general anaesthesia (Dex).

*Cum*. cumulative dose in all patients, *Dex* Dexmedetomidine, *IQR* Interquartile range. *n* (%) number of patients who received the drug or transfusion at least once (expressed as percentage), *RBC* red blood cell, *TIVA* totally intravenous anaesthesia.

^a^Categorical variables analysed by Chi^2^ test.

^b^Continuous variables analysed by Mann–Whitney U test.

### AChE and BChE activities decrease postoperatively in placebo treated patients, but remain stable in dexmedetomidine treated patients

**Figure 2:**
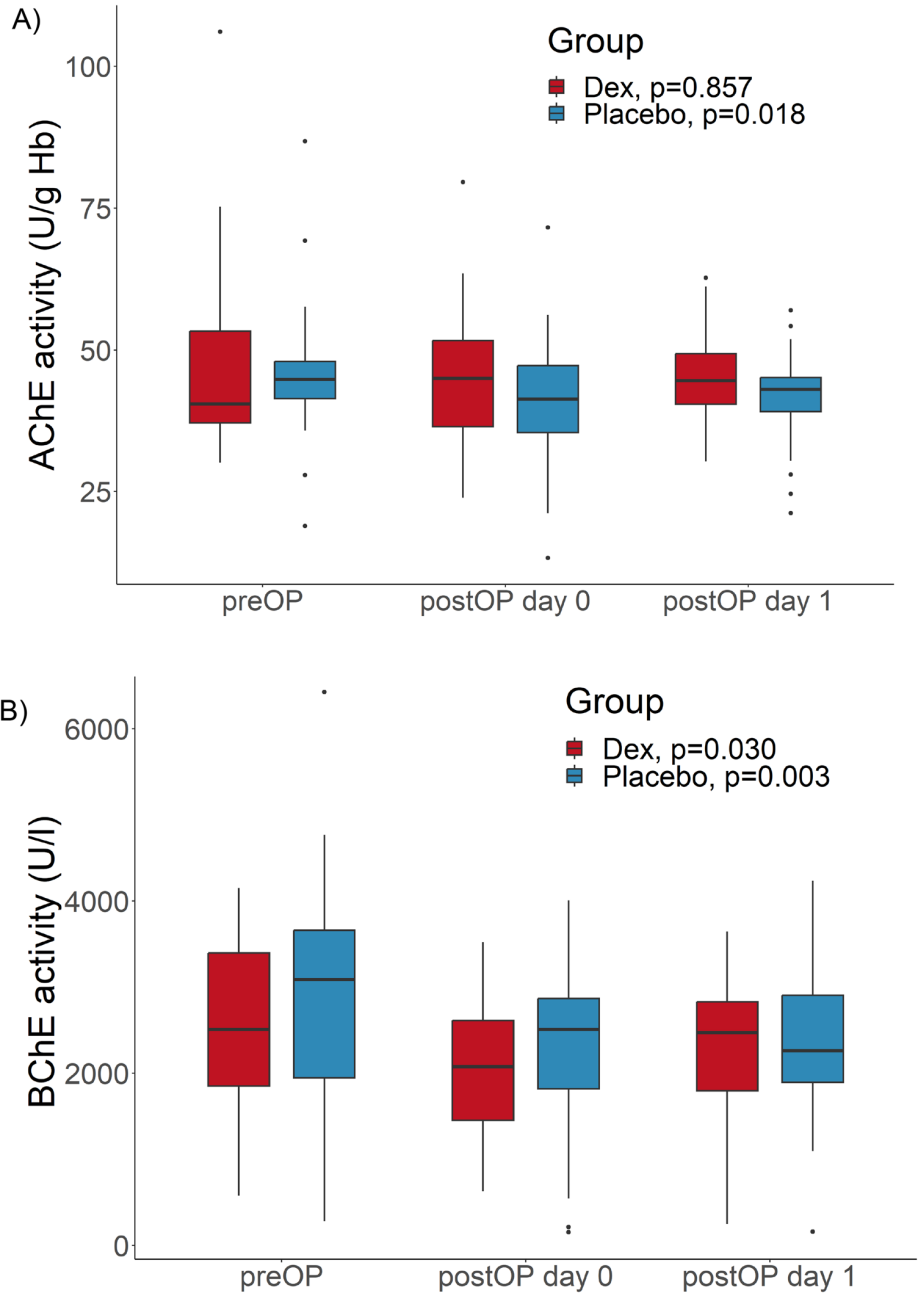
Activities of cholinesterases. (**A**) Activities of acetylcholinesterase (AChE) (**B**) Activities of butyrylcholinesterase (BChE) before (preOP), 15 min after (postOP day 0) and one day after surgery (postOP day 1) compared between patients receiving standard general anaesthesia (Placebo, blue, n = 30) or general anaesthesia combined with perioperative dexmedetomidine (Dex, red, n = 26). The Boxplots show median and IQR (lower and upper hinges). *P*-values compare values of all three points in time within each group, using the Friedman rank analysis.

Enzyme activity of AChE in the placebo group changed significantly over the course of the three points in time preoperatively, directly after surgery and on postoperative day one, with an overall decline in activity (Fig. 2). In contrast, in the dexmedetomidine group, AChE activity did not change significantly in the course of these three points in time, despite a tendency of upregulated enzyme activity. The course of BChE activity showed a significant change in the placebo-treated patients and indicated a postoperative decline. In the dexmedetomidine group, BChE values decreased directly after surgery and increased again on postoperative day one. Nonetheless, this change in enzyme activity was significant over all three points in time.

Regarding between-group comparisons, neither preoperative baseline values nor postoperative values of AChE and BChE activity showed any significant difference between dexmedetomidine and placebo treated patients (Table 3).

**Table 3:** Activities of acetylcholinesterase (AChE) and butyrylcholinesterase (BChE) before (preOP), 15 min after (postOP day 0) and one day after surgery (postOP day 1) compared between patients receiving standard general anaesthesia (Placebo, n = 30) or general anaesthesia combined with perioperative dexmedetomidine (Dex, n = 26).

AChE (U/g Hb)	preOP	postOP day 0	postOP day 1
Placebo	44.80 [40.88; 48.05]	41.35 [33.43; 47.50]	43.05 [38.08; 45.63]
Dex	40.50 [36.78; 54.90]	45.00 [35.93; 53.30]	44.60 [40.23; 49.73]
*P*-value	0.388	0.194	0.257

BuChE (U/l)	preOP	postOP day 0	postOP day 1
Placebo	3087.9 [1871.4; 3721.2]	2511.7 [1686.1; 2889.9]	2260.1 [1876.7; 2927.1]
Dex	2511.8 [1766.2; 3418.6]	2074.4 [1436.2; 2618.1]	2474.4 [1701.4; 2874.7]
*P*-value	0.194	0.293	0.908

Tables show medians with interquartile ranges. *P*-values compare values at individual points in time between both groups with the Mann–Whitney U test.

### Cholinesterase activities in patients with or without delirium

**Figure 3:**
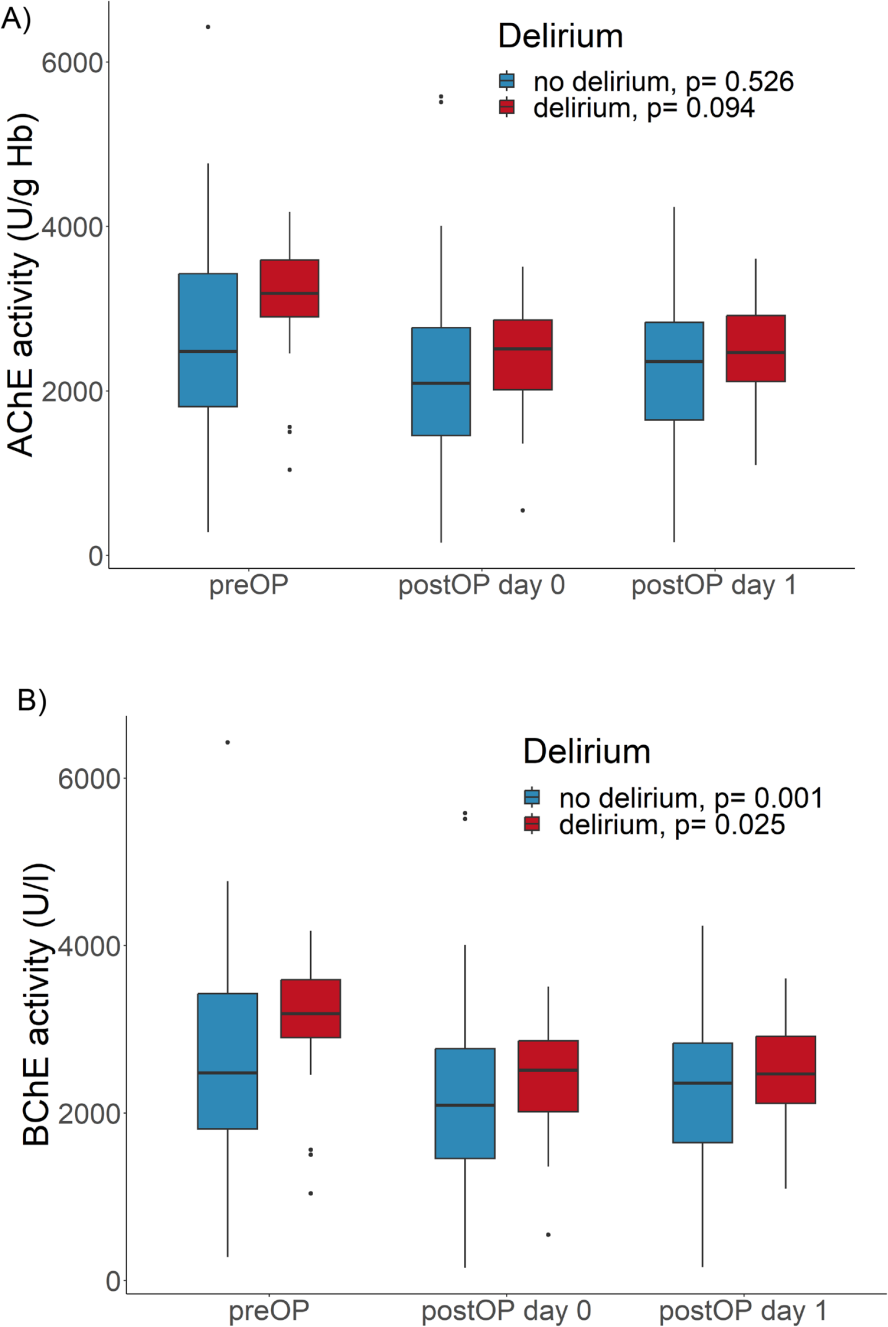
Activities of cholinesterases. (**A**) Activities of acetylcholinesterase (AChE) (**B**) Activities of butyrylcholinesterase (BChE) before (preOP), 15 min after (postOP day 0) and one day after surgery (postOP day 1) compared between patients without delirium (no delirium, blue, n = 42) or patients with delirium (delirium, red, n = 14). The Boxplots show median and IQR (lower and upper hinges). *P*-values compare values of all three points in time within each group, using the Friedman rank analysis.

AChE activities tends to decrease postoperatively in patients with delirium more, but remain stable in patients without delirium, while BChE activities decrease postoperatively in both, patients with and without delirium (Fig. 3).

## Discussion

The present study shows for the first time in human subjects that dexmedetomidine combined with general anaesthesia leads to a stable perioperative AChE activity in blood of patients with major abdominal or cardiac surgery. In contrast, general anaesthesia alone leads to a significant decrease in enzyme activity.

This is in line with findings of Nemoto and colleagues who studied the effect of intravenous propofol, midazolam and dexmedetomidine on the extracellular concentration of acetylcholine in the cerebral cortex of rats^60^. Propofol and midazolam led to a decrease in acetylcholine release, whereas they found no significant change after administration of dexmedetomidine. In contrast, dexmedetomidine-mediated activation of α2-adrenoceptors in animal models of neuropathic pain enhances analgesia by increasing ACh release in the spinal cord, whereas stimulation of α2-adrenoceptors results in inhibition of acetylcholine release under normal circumstances^61^. Kho et al. investigated the effect of dexmedetomidine on acetylcholine expression and AChE activity in the cortex, hippocampus, and spleen in rats with LPS-induced inflammation. In the spleen, dexmedetomidine exerts an activating effect on AChE and prevents an LPS-induced decrease in AChE activity. Additionally, the dexmedetomidine-mediated increase in AChE did not correlate with a decrease in ACh, nor did it coincide with an increase in apoptosis markers, indicating that AChE may also have played other roles than ACh hydrolysis^41^. In fact, Soreq and colleagues pointed out, that AChE also has “non-classical” effects, such as neuritogensis, synaptogenesis as well as haematopoiesis and thrombopoiesis^62^. In a small clinical study of 48 patients who had undergone major pancreatic surgery with or without splenectomy, the postoperative decrease in BChE activity was attenuated in patients with splenectomy compared to patients with a preserved spleen^63^. This highlights the integral role of the spleen in the CAIP and is consistent with the experimental work of Hoover and colleagues. They found that cholinergic function in the spleen is enhanced in sepsis by mechanisms of gene expression and reduction in cholinesterase activity, which facilitates cholinergic transmission^64^. Furthermore, they hypothesised that because choline is also a selective agonist for α7nAChRs and because the spleen lacks high-affinity choline transporters, the resulting prolonged presence of extracellular choline enhances cholinergic signalling in the spleen^65^.

We observed that patients treated with dexmedetomidine had stable AChE and rapidly recovering BChE activity, whereas they showed a lower incidence of POD in the primary study^8^. This may be related to the anti-inflammatory and immunomodulatory properties of dexmedetomidine. Deregulation of the CAIP caused by cholinergic deficiency leads to increased inflammation with neuroinflammation, which in turn promotes the development of POD^31–33^.

AChE plays a key role in cholinergic transmission by hydrolysing ACh at maximum rate, thereby maintaining not only cholinergic transmission but also the recycling process of ACh synthesis, where the absence of one component can lead to cholinergic dysfunction. Early animal studies demonstrated a central decrease in ACh synthesis and release in old age^66,67^. The present analysis focuses on elderly surgical patients with predominantly abdominal or high-risk cardiac surgery who are particularly affected by severe surgical trauma and long surgical duration with correspondingly high anaesthetic and opioid requirements. These factors influencing cholinergic transmitter balance, coincide with a predisposed population of high susceptibility to postoperative complications and may promote the development of POD. Furthermore, in the postoperative course of the CESARO study, a lower recovery of AChE activity was observed in the older subjects than in the younger ones. The authors argue that this effect is related to a decrease in regulatory capacity of the cholinergic system with age^51^.

BChE acitivity continuously decreased in placebo treated patients, whereas in dexmedetomidine treated patients, there was an increase on the first postoperative day compared to directly postoperative. On the one hand, this might be related to the fact that BChE activity is more susceptible to confounding factors, such as the outcome of hemodilutional cardiopulmonary bypass or malignancy^42^. On the other hand, BChE activity also responds rapidly to inflammation with a decrease in activity^55^. Characteristically for a negative acute phase protein, BChE activity has repeatedly been shown to be a prognostic biomarker for disease severity in acute inflammatory conditions such as sepsis, trauma, and burns^55–58,68^. Thereby, a clear pattern of BChE activity was observed: After an initial decrease in BChE activity, a sustained low level of activity was associated with higher morbidity and mortality, whereas an increase in activity during the course of the disease was considered as a sign of recovery and a better outcome^56,57^. However, because in the present study cholinesterases were measured only up to the first postoperative day, it can be merely speculated how BChE would have developed at later points in time in patients treated with dexmedetomidine.

Regarding the underlying physiological mechanism, Xiang and colleagues demonstrated that intraperitoneal administration of dexmedetomidine in mice augmented the electric activity of the cervical vagus nerve^27^. Furthermore, dexmedetomidine led to reduced levels of systemic inflammatory cytokines (TNF-α, IL-1β, IL-6). Interestingly, the anti-inflammatory effect of dexmedetomidine was also abolished by the administration of an antagonist at the α7nAChR, suggesting that dexmedetomidine has an influence on the CAIP. In similarly designed animal models, dexmedetomidine also reduced pro-inflammatory cytokine levels directly in the brain of rats^69^ and protected against cognitive dysfunction following systemic inflammation^20,70^. Moreover, Ohta and his colleagues investigated the anti-inflammatory effect of dexmedetomidine in patients with sepsis^71^. Patients treated with dexmedetomidine in addition to standard intensive care had significantly lower levels of the inflammatory markers CRP and PCT.

Although the few publications on the role of cholinesterases in POD yield inconsistent findings, they highlight the close relationship between the cholinergic system, perioperative inflammation and POD. In a cohort observational study of patients undergoing arthroplasty surgery, Cerejeira and colleagues found a correlation between low preoperative esterase activity and a change in blood CRP concentration^47^. Strikingly, this correlation was only found in patients with delirium but not in patients without POD. Additionally, a study by Adam and colleagues of patients undergoing cardiac surgery also found that POD correlated with decreased AChE activity before and after surgery, confirming that pre-existing altered cholinergic function may be a risk marker for POD^46^. Consistent with these reports, a recently published prospective observational study investigated the relationship between POD and cholinesterases in elderly non-cardiac surgery patients^48^. Both cholinesterases were found to be significantly lower postoperatively in patients with POD compared to those without POD. The authors of that study speculate that postoperatively decreased AChE and BChE activity in POD patients might be a response to already decreased acetylcholine levels before surgery. However, we cannot confirm this with our analysis, as the preoperative values in both groups are within the reference range. Nevertheless, it is quite interesting that the baseline values of both cholinesterase activities were lower in the dexmedetomidine than in the placebo group, although this is not statistically significant. Given the studies suggesting that low cholinesterase activity preoperatively increases the risk of POD^46–51^, one could speculate whether, without treatment with dexmedetomidine, the incidence of delirium might have been even higher in this group than in the placebo group.

In contrast to our results, a study in cardiac surgery patients showed no difference in postoperative measurements of AChE and BChE between patients with and without POD^72^. In addition, Mueller and her colleagues reported upregulated AChE in patients with POD and considered this as a compensatory mechanism for cholinergic deficit to provide new metabolites for acetylcholine synthesis^51^.

### Strengths and limitations

This secondary analysis was conducted using a randomised, double-blind, placebo-controlled trial. To our knowledge, this is the first clinical trial to investigate the influence of intra- and postoperatively administered dexmedetomidine on the perioperative course of AChE and BChE. Overall, the results of the present study strengthen the role of the cholinergic system in delirium pathogenesis and at the same time suggest a regulatory effect of dexmedetomidine on the cholinergic system.

The present study did not show a significant difference in postoperative changes of AChE activity between dexmedetomidine and placebo treated patients. This may be due to a relatively small population size. Administrative and technical limitations led to incomplete follow-up measurements in some subjects. Moreover, the trial was powered for the primary outcome of POD incidence on the one hand, on the other hand, baseline values of both groups showed large interquartile ranges pointing towards high variation. We used a portable point-of-care photometer, which was originally designed for the analysis of ChE inhibition in organophosphate poisoning.

Cholinesterase activity is subject to a circadian rhythm^73^. This is a matter of ongoing research, also in the context of drugs for treatment of Alzheimer disease and optimal timing for their intake^74^. In our study, preoperative values were measured either on the evening before surgery or in the morning before surgery.

Therefore, preoperative values are likely influenced by circadian fluctuation. In our opinion, strict timing of AChE and BChE measurements could yield more homogenous results and significant between-group differences. Considering the existing difference between the groups in terms of their baseline esterase activities (AChE: 44.80 vs. 40.5 and BChE: 3088 vs. 2512), we have to assume that the preoperative values were probably influenced by circadian fluctuations. In our opinion, strict timing of AChE and BChE measurements could lead to more homogeneous baseline activity values and significant differences in activity progression between the groups.

A further subdivision of the groups into patients who actually developed delirium or remained delirium-free was not made due to the already very small group sizes (a total of 14 with delirium, 3 of which were in the dexmedetomidine group). However, such an analysis would probably be helpful with a larger population to obtain more informative results. There is also limited interpretation of cholinesterase activities in the groups with and without delirium up to the first postoperative day (Fig. 3), as postoperative delirium is the very disease that dexmedetomidine is supposed to ameliorate.

It should also be considered that our patients were recruited from two distinct populations: major abdominal surgeries and coronary artery bypass graft surgeries. One could suppose that these populations are distinct from one another, for instance in regard to perioperative blood loss and subsequent transfusions which is usually higher in cardiac surgeries. It has already been demonstrated by John and her colleagues that these factors alter the course of perioperative cholinesterases in patients who underwent cardiac surgery^75^.

As pro-inflammatory markers such as CRP were not recorded in the primary database, we could not analyse the relationship between cholinesterase activity, inflammatory markers and dexmedetomidine.

There is only limited literature about the regulation of cholinesterases, which makes it challenging to examine homogenous populations. A further limitation results from the lack of comparable studies on the effect of dexmedetomidine on cholinesterases. Accordingly, many reference studies have a different experimental design and comparability to our study is limited.

Therefore, we regard this secondary analysis rather as hypothesis-generating and we advocate for further investigations that are statistically powered for this question and that examine homogenous populations.

## Conclusion

The present secondary analysis of a randomised controlled trial points towards a possible association between the perioperative use of dexmedetomidine and the regulation of cholinesterase activities. Patients treated with dexmedetomidine had stable perioperative AChE activity levels and the initial postoperative decline in BChE activity recovered rapidly, whereas placebo-treated patients showed a steady postoperative decline in both enzyme activities. Since neuroinflammation plays a pivotal role in the pathogenesis of POD, our data makes it tempting to speculate that dexmedetomidine may augment the CAIP by acting on cholinesterase activity, thereby alleviating POD.

## Data Availability

The data presented in this study are available on reasonable request from the corresponding author. The data are not publicly available due German data privacy protection regulations for clinical trials according to the Federal Institute for Drugs and Medical Devices.
